# Vascular Interventions and Surgery in Trauma Audit (VISTA)

**DOI:** 10.1093/bjsopen/zrad012

**Published:** 2023-04-13

**Authors:** Katherine-Helen Hurndall, Hannah Merriman, Robert Leatherby, Simon Glasgow, Ross Davenport

**Affiliations:** Centre for Trauma Sciences, Blizzard Institute, Queen Mary University of London, London, UK; Department of Vascular Surgery, The Royal Free Hospital, London, UK; Department of Vascular Surgery, Glenfield Hospital, Leicester, UK; Department of Vascular Surgery, Royal Bournemouth Hospital, Bournemouth, UK; Imperial Vascular Unit, Imperial College Healthcare NHS Trust, St. Mary’s Hospital, London, UK; Centre for Trauma Sciences, Blizzard Institute, Queen Mary University of London, London, UK

*Dear Editor*

In the UK, major trauma remains the leading cause of death in the first four decades of life, with exsanguination being the primary cause of preventable death^1,2^. Major trauma also represents the most common cause of limb loss amongst young adults^1,2^. Despite these patients having a mortality rate in excess of 10 per cent, there is no national registry of incidence, procedural key performance indicators or clinical outcomes. Whilst the Trauma Audit and Research Network (TARN) collects data pertaining to vascular injury, it lacks the granularity to allow detailed evaluation of management strategies, patient pathways and outcomes^3^. The National Vascular Registry (NVR) excludes patients with traumatic vascular injury. Accurate and comprehensive data collected across the whole patient pathway is critical to understand the current incidence, contemporary management and outcomes associated with vascular trauma in the UK. Due to the relatively low incidence of vascular injury, a large, multicentre audit is required to determine the national burden of vascular injury and current practice.

The primary aim of the Vascular Interventions and Surgery in Trauma Audit (VISTA) is to determine the current UK incidence of vascular trauma and compare management with guidelines produced by the American Association of Surgery for Trauma and the Eastern Association for the Surgery of Trauma^4,5^. The secondary aims are to describe the associated patient outcomes and current patient pathways.

VISTA is a national, multicentre, prospective, collaborative, trainee-led audit by the National Trauma Research and Innovation Collaborative (NaTRIC) and the Vascular and Endovascular Research Network (VERN)—the first such collaboration between these two research networks (full protocol in *Appendix S2*). All major trauma centres in the UK were invited to participate in VISTA; 30 of 31 centres have been recruited and commenced data collection in March and April 2022 for a consecutive 6-month period (condensed data points *Table 1*). All patients with radiologically, and/or intraoperatively proven vascular injury in the UK were eligible for inclusion. Patients with isolated intracranial and/or coronary vessel injuries, iatrogenic injuries, isolated injuries to the superficial venous system and isolated injuries to vessels distal to the popliteal trifurcation or brachial bifurcation were excluded. The end of patient level follow-up is defined as the point at which the patient either dies, is discharged from hospital or reaches day 30 of their inpatient stay. Data were collected by each recruited site and entered into a centrally managed database using the Research Electronic Data Capture (REDCap) system (*Table S1*). Each centre needed local approval from their audit and Caldicott guardian before commencing data collection but ethics approval was not required. The results of the study will be disseminated to each site, submitted for presentation at academic conferences and will be submitted for peer-reviewed publication. A collaborative authorship model will be employed for both presentation and publication (full protocol in *Appendix S2*).

**Table 1:** Complete list of data points to be collected

Data field	Data specifics
Patient demographics	Sex
	Age (in years)
	Ethnicity
	Co-morbidities—smoking status, diabetes, hypertension, ischaemic heart disease, chronic kidney disease, peripheral vascular disease
Injury data	Type of injury—blunt *versus* penetrating
	Mechanism of injury
	Injury sustained
	Any associated injuries?
	Abbreviated injury scale
	Injury severity score
	Observations and blood gas on arrival in the ED
	Prehospital + ED intervention—tourniquet, blood products, tranexamic acid, thoracostomy/thoracotomy
	Grade of shock on arrival in ED
Site data	Location of presenting hospital—rural or urban?
	Transferred out?
	Transfer destination (vascular centre, major trauma centre)
	Time from presentation to transfer (mins)
	Reason for transfer + any delay
Management data	Method of diagnosis—imaging modality *versus* surgery
	Time from presentation to intervention (mins)
	Intervention performed
	Most senior grade of surgeon
	Primary operator specialty
Operative findings	Record anatomical level of injury
	Confirm injury sustained
	Materials used in repair
	Primary amputation?
Outcome data	Total length of stay (days)
	Length of critical care stay (days)
	Death at 30 days
	Amputation rate at 30 days
	Complications
	Return to theatre
	Discharge destination

ED, emergency department. See Supplementary material for more detail.

VISTA is an innovative project that will capture important data on the state of vascular trauma care in the UK and hopes to inform improvements in vascular trauma management. Having recruited all but one major trauma centre in the UK with 309 patients entered into REDCap to date (December 2022), the data will be widely applicable, allowing comparison of current management and will identify any geosocial disparities that may exist. As no current UK-specific guidelines exist on the management of vascular trauma, it is expected that the results of this study will inform the design of future studies and development of national guidelines.

## Collaborators

G. K. Ambler (University of Bristol, Bristol UK); L. Hitchman (Hull-York Medical School, Hull, UK); R. A. Benson (University of Otago, Department of Academic Surgery, Christchurch, New Zealand); P. Birmpili (Hull York Medical School, Hull, UK); R. H. J. Blair (Royal Victoria Hospital, Belfast, UK); D. C. Bosanquet (Aneurin Bevan University Health Board, Newport, UK); N. Dattani (University Hospital of Leicester, Leicester, UK); G. Dovell (University of Bristol, Bristol, UK); B. L. Gwilym (Aneurin Bevan University Health Board, Newport, UK); M. Machin (Imperial College, London, UK); S. Nandhra (Northern Vascular Centre, Freeman Hospital, Newcastle, UK); S. Onida (Imperial College, London, UK); J. Shalhoub (Imperial College, London, UK); A. A. Singh (University of Cambridge, Cambridge, UK); A. Saratzis (University of Leicester, Leicester, UK); J. Wohlgemut (Centre for Trauma Sciences, Blizard Institute, Queen Mary University, London, UK); M. Marsden (Centre for Trauma Sciences, Blizard Institute, Queen Mary University, London, UK); J. Ross (Centre for Trauma Sciences, Blizard Institute, Queen Mary University, London, UK); P. Vulliamy (Centre for Trauma Sciences, Blizard Institute, Queen Mary University, London, UK); A. Rossetto (Centre for Trauma Sciences, Blizard Institute, Queen Mary University, London, UK); R. Carden (Centre for Trauma Sciences, Blizard Institute, Queen Mary University, London, UK).

## Supplementary Material

zrad012_Supplementary_Data

## Data Availability

The data used in this paper is widely accessible to the public and may be accessed via the references at the end of the article.
