# Supplementary material for: Vascular Interventions and Surgery in Trauma Audit (VISTA)
Source: BJS Open. 2023 Apr 13;7(2):zrad012. doi: 10.1093/bjsopen/zrad012 (PMC10101046; doi:10.1093/bjsopen/zrad012)
Supplement: zrad012_Supplementary_Data [file zrad012_supplementary_data.docx]

**Title**

The Vascular Interventions and Surgery in Trauma Audit (VISTA)

**Authors**

Katherine-Helen Hurndall^1,2^ Hannah Merriman^3^, Robert Leatherby^4^, Simon Glasgow^5^, Ross Davenport^1^

^1^Centre for Trauma Sciences, Blizzard Institute, Queen Mary University of London

^2^ The Royal Free Hospital, Department of Vascular Surgery, London

^3^Glenfield Hospital, Department of Vascular Surgery, Groby Road, Leicester

^4^Royal Bournemouth Hospital, Department of Vascular Surgery, Castle Lane East, Bournemouth

^5^Imperial Vascular Unit, Imperial College Healthcare NHS Trust, St. Mary’s Hospital, London

**Corresponding author:**

Katherine-Helen Hurndall, The Royal Free Hospital, Pond St, London. NW3 2QG

[khurndall@doctors.org.uk](mailto:khurndall@doctors.org.uk)

ORCID ID - https://orcid.org/0000-0002-9161-968X

Twitter - @KHurndall93, @2022VISTA

**Supplementary Materials - Index**

| **Supplementary Appendixes** |  |
| --- | --- |
| Appendix S1. List of Collaborators | *page 2* |
| Appendix S2. Study Protocol for the Vascular Interventions and Surgery in Trauma Audit (VISTA) | *pages 3 - 10* |
| **Supplementary Figures and Tables** |  |
| Supplementary Table 1 – List of Datapoints | *pages 11- 12* |
|  |  |

**Supplementary Appendixes**

**Appendix S1. List of Collaborators**

**Vascular and Endovascular Research Network (VERN) Executive Committee:**

Graeme K Ambler (University of Bristol, Bristol UK)

Louise Hitchman (Hull-York Medical School, Hull, UK)

Ruth A Benson (Universitu of Otago, Department of Academic Surgery, Christchurch, New Zealand)

Panagiota Birmpili (Hull York Medical School, Hull, UK)

Robert H J Blair (Royal Victoria Hospital, Belfast, UK)

David C Bosanquet (Aneurin Bevan University Health Board, Newport, UK)

Nikesh Dattani (University Hospital of Leicester, Leicester, UK)

George Dovell (University of Bristol, Bristol, UK)

Brenig L Gwilym (Aneurin Bevan University Health Board, Newport, UK)

Matthew Machin (Imperial College, London, UK)

Sandip Nandhra (Northern Vascular Centre, Freeman Hospital, Newcastle, UK)

Sarah Onida (Imperial College, London, UK)

Joseph Shalhoub (Imperial College, London, UK)

Aminder A Singh (University of Cambridge, Cambridge, UK)

Athanasios Saratzis (University of Leicester, Leicester, UK)

**National Trauma Research and Innovation Collaborative (NaTRIC) Executive Committee:**

Jared Wohlgemut (Centre for Trauma Sciences, Blizard Institute, Queen Mary University, London, UK)

Max Marsden (Centre for Trauma Sciences, Blizard Institute, Queen Mary University, London, UK)

Jennifer Ross (Centre for Trauma Sciences, Blizard Institute, Queen Mary University, London, UK)

Paul Vulliamy (Centre for Trauma Sciences, Blizard Institute, Queen Mary University, London, UK)

Andreas Rossetto (Centre for Trauma Sciences, Blizard Institute, Queen Mary University, London, UK)

Richard Carden (Centre for Trauma Sciences, Blizard Institute, Queen Mary University, London, UK)

**Appendix S2. Study Protocol for the Vascular Interventions and Surgery in Trauma Audit (VISTA)**

**Abstract**

Introduction

Vascular trauma kills. Following traumatic injury, exsanguination is the primary cause of preventable death in a primarily healthy and young population. Trauma patients with vascular injury have a mortality rate in excess of 10% and yet there is no national registry of incidence, procedural key performance indicators or clinical outcomes. The primary aim of VISTA is to describe the incidence of vascular trauma and to determine whether the contemporaneous management of vascular injury aligns with established guidelines published by the American Association of Surgery for Trauma (AAST) and Eastern Association for the Surgery of Trauma (EAST). The secondary aim is to describe the associated patient outcomes.

Methods and Analysis

This national, multicentre, prospective, collaborative, trainee-led audit will be delivered in conjunction with the National Trauma Research and Innovation Collaborative (NaTRIC) and the Vascular and Endovascular Research Network (VERN). Participating UK major trauma centres will identify all patients presenting with confirmed vascular trauma on imaging or intraoperatively during a consecutive six-month period. Data will be entered into a centrally managed database using the Research Electronic Data Capture (REDCap) system.

Ethics and Dissemination

The project will be registered with local audit departments at all participating centres. Research ethics approval is not required as data is routinely collected as part of clinical practice and will not change the treatment patients receive. The VISTA committee will oversee all registrations, approvals and data collection.

**Introduction**

In the United Kingdom, major trauma is the leading cause of death in the first four decades of life and the most common cause of limb loss amongst young adults^1-3^. Patients with vascular trauma suffer a high mortality rate and for those who survive, there is significant associated morbidity^4^. These primarily young, healthy patients utilise significantly more hospital resources compared to trauma patients without vascular injury, requiring up to a third of all blood transfusions and a six times longer inpatient admission^5^. Whilst international guidelines exist for the management of vascular trauma^6-11^, there are no United Kingdom (UK) specific guidelines.

Currently, there is limited data on the incidence, management and outcomes of patients presenting with vascular trauma in the UK. The Trauma and Audit Research Network (TARN) collects data pertaining to vascular injury however, it lacks the granularity to allow significant evaluation of management strategies, patient pathways and patient outcomes^12^. The National Vascular Registry (NVR), which records data relating to vascular interventions, excludes patients with traumatic vascular injury. Accurate and comprehensive data collected across the whole patient pathway is critical to understand the current incidence, contemporary management and outcomes associated with vascular trauma in the UK. Due to the relatively low incidence of vascular injury, a large, multicentre service evaluation is required to understand the national burden of vascular injury and current practice.

The Vascular and Endovascular Research Network (VERN) and the National Trauma and Research and Innovation Collaborative (NaTRIC) are both well-established UK trainee-led research collaboratives. Their multidisciplinary structures facilitate collaborative working between surgeons, nurses, therapists and paramedics at a national level. They have both successfully delivered multiple, collaborative, multicentre audits and service evaluations at low cost^13-15^. The primary aim of VISTA is to describe the UK incidence of vascular trauma and to determine whether the contemporaneous management of vascular injury aligns with established guidelines published by the American Association of Surgery for Trauma (AAST) and Eastern Association for the Surgery of Trauma (EAST*).* The secondary aim is to describe the associated outcomes for patients who have sustained vascular trauma, specifically mortality, length of stay and amputation rate (primary and delayed).

**Methods and Analysis**

*Design*

A national, multicentre, prospective audit of UK practice, supported and disseminated via NaTRIC and VERN.

*Setting*

All major trauma centres (MTCs) in the UK were invited to participate in the VISTA project. 30 out of the 31 MTCs across Scotland, Wales, England and Northern Ireland have been recruited by the VISTA central committee, with support from NaTRIC and VERN.

*Time Frame*

The VISTA audit was officially launched on 1^st^ March 2022 with recruitment of new centres terminating on 11^th^ April 2022. Centres commenced data collection in March and April 2022, for a six month period. Data from consecutive vascular trauma patients that meet the inclusion criteria will be collected prospectively during this period.

The end of patient level follow-up is defined as the point at which the recruit patient either dies, is discharged from hospital, or reaches day 30 of their inpatient stay.

*Participants*

The audit will enrol consecutive patients presenting with vascular trauma to recruited MTCs. Vascular trauma is defined as injury to one or more named extra-cranial and extra-coronary vessels.

Inclusion Criteria:

- All patients with radiologically, or surgically proven vascular trauma (as defined above) in the UK.

Exclusion Criteria:

- Isolated intracranial and/or coronary vessel injuries
- Iatrogenic injuries e.g. trochar injuries
- Isolated injuries to the superficial venous system
- Isolated injuries to vessels distal to the popliteal trifurcation or brachial bifurcation
- Trauma patients without vascular injury

*Patient Identification and Data Collection/ Management*

Case identification will be the responsibility of the local study team (Table 1). Surgical and/or radiology trainees at each site will review all trauma cases within the previous 24 hour period (or if over a weekend or bank holiday, as soon as reasonably possible) to identify if any vascular injury was diagnosed and/or intervened upon. Patients will be pseudonymised at each participating centre. It will be the responsibility of the local audit team to enter the patient information into the central REDCap database. Liaison with local TARN coordinators will help to minimise data loss. The full data points to be collected are listed in Table 2.

It is essential that each recruited MTC maintains a local database, securely stored on an NHS computer only, to link the local/ national ID numbers to the REDCap IDs. This will allow outcomes to be recorded and subsequently entered into the REDCap database. It is the local site’s principal investigator’s responsibility to ensure this is completed and stored securely.

Data will be obtained from patients’ notes and electronic records, - including (but not limited to) emergency department records, discharge letters, imaging reports, blood reports and operation notes. No direct changes to standard patient care will occur as a result of this audit.

Data will be collected and managed using the Research Electronic Data Capture (REDCap – Vanderbilt, Nashville, USA) electronic data capture tool (hosted at Newcastle University Hospitals). Anonymised data will be analysed by the VISTA central committee by amalgamation of the individual site data. Patients will be included if 80% or more of individual patient’s data is recorded.

*Primary Outcome*

The primary outcome is to compare current UK incidence and management of vascular trauma with the established guidelines produced by the AAST and EAST^6-11^.

*Secondary Outcomes*

The secondary outcomes include:

- Observed patterns and mechanisms of injury (including concomitant injuries)
- Number of patient’s transferred from spoke hospital to MTC
- Transfer time from spoke hospital to MTC
- Utilisation and choice of imaging modalities
- Length of ICU/HDU admission
- Total length of hospital stay
- Return to theatre rate
- 30 day mortality rate
- Rate of complications (Table 2)
- Rate of limb salvage
- Discharge destination

*Data Completeness and Validation*

The importance of complete data is acknowledged. Cases which have less than 80% data completeness will be excluded from analysis.

At the end of the data collection period, 50% of the cases for each site will be randomly selected and a random 20% of datapoints shall be returned to the respective site to confirm data accuracy, as is standard with collaborative audits (16-17). Compliance with this process is mandatory and data must be 95% accurate Any site reporting less than 95% accuracy will be required to validate a further 20% of their cases. The lead investigator for each site will be required to undertake this process and report back to the VISTA central team.

*Statistical Analysis*

Based on TARN data, a 6-month data collection period is estimated to capture vascular trauma data on approximately 400 cases. Analyses will be performed to determine the incidence of, and approaches to, management of vascular trauma in the UK. Analysis will also examine secondary outcomes.

Data will be checked for normality and the appropriate statistical tests will be selected. Data will be reported as means with standard deviations (SD) for normally distributed continuous data, and medians with interquartile ranges (IQR) for non-normally distributed continuous data. Categorical data will be presented as frequencies and percentages. Where comparisons between groups are made, the *t*-Test and Mann-Whitney U Tests will be used for normally and non-normally distributed continuous data, respectively. Categorical data will be analysed using Pearson’s Chi-squared tests. Statistical analysis and graph design will be performed using appropriate statistical analysis software. A p-value of <0.05 will be considered statistically significant.

*Centre Eligibility and Team Roles*

All UK major trauma centres with an inpatient 24/7 arterial service are eligible to enrol in the project. Each centre will have a designated consultant principal investigator who will be the local lead for VISTA. In addition, each site will endeavour to enrol 1 or more surgical or radiology trainees as site “trainee leads” to perform data collection. Non-UK centres are not eligible for enrolment into VISTA.

The principal investigator will ensure local audit approval is obtained, promote local engagement with the VISTA project, hold overall responsibility for data collection and upload and facilitate departmental/ local presentation of results.

**Ethics and Dissemination**

*Ethics and Registration*

The NHS Health Research Authority (HRA) decision-making tools, “Is My Study Research?”^18^ and “Do I need NHS Research Ethics Committee approval?”^19^ were utilised to determine that this audit does not require approval from an NHS Research Ethics Committee.

Each contributing site will need to register VISTA with their audit department and seek Caldicott Guardian approval. A named consultant at each site will act as Principal Investigator and will be responsible for supervising the project locally.

*Dissemination and Authorship*

Study results will be disseminated locally at each site, published on social medical and submitted for presentation at national and international academic conferences. A manuscript will be prepared for peer-reviewed publication. The central VISTA team will be responsible for both presentations and publications. For both presentation and publication, a collaborative authorship model will be used. Criteria to quality for collaborative authorship are defined as:

1. Had a significant role in the set up and management of the VISTA audit; including audit department registration, creation of a data collection team, and engagement with the VISTA committee to ensure timely upload of data (with validation as required).

OR

1. Captured sufficient data to warrant authorship – this would be the equivalent of collecting baseline and follow up data on approximately 12 patients, although it is appreciated individuals may participate in only baseline data collection or only follow up data capture. Data collection is expected to be complete (>80% variables completed) and submitted within 9 months of starting data collection.

OR

1. Provide oversight and support as detailed in the “Centre eligibility and team roles” section (for principal investigators).

AND

1. Review and approve any resultant manuscript(s) for submission to a peer-reviewed journal.

The corresponding author will take primary responsibility for communication with the journal throughout the submission process.

The anticipated number of audit team members per centre is: 1 principal investigator + 2 other team members (1 vascular/ trauma-interested surgical trainee, 1 radiology trainee). If centres include more than 2 additional team members, it is expected that allied health professionals and/or medical students are included.

Each site may also choose to present their individual findings at local/ departmental meetings. Sites will not be analysed individually by the central team.

**Discussion**

VISTA is a national, multicentre audit of practice, to enable detailed investigation of the incidence, contemporary management and outcomes of vascular trauma across the UK and compare them to current, available guidelines. A multicentre approach will enable the rapid collection of significant volumes of data and facilitate meaningful statistical analysis. By recruiting patients from the majority of MTCs in the UK, the results will be geographically representative.

This project is the first of its kind, collecting detailed national data on the pattern of injury in vascular trauma and evaluating the specific management techniques used. The data collected by TARN focuses primarily on survival data and allows comparison of outcomes between MTCs in the UK. The survival data published relates to the overall injury severity score (ISS) for the patient and it is not possible to accurately determine the cause of death for patients. Whilst the data does record if an operation was performed, it does not provide sufficient detail as to the procedure(s) performed or any information on the operative findings. It does not explain reasons for delayed operative intervention or conservative management. As such, the data lacks the granularity to allow for focused analysis of vascular injury and the related patient outcomes.

No current UK-specific guidelines exist on the management of vascular trauma. It is expected that the results of this study will allow comparison of current management techniques and inform either further study design or the development of national guidelines.

Limitations of this audit include difficulties in confirming that cases are truly consecutive and data validation. Another potential limitation is lack of data to allow or sub-group analysis. However, VISTA will still be able to narratively explore correlations and suggest areas for future research.

National, multicentre audits are a well-recognised method of collecting large volumes of data to facilitate the development of national guidelines and drive enhancements in clinical practice. VISTA is an innovative project that will capture important data on the state of vascular trauma care in the UK and bring about improvements in the management of vascular trauma across the UK whilst helping to identify any geo-social disparities that may exist.

**References**

1. DfT. Reported road casualties: Great Britain 2010—annual report.

2. Office for National Statistics. Deaths registered in England and Wales: 2020. onsgovuk [Internet]. 2021 Jul 5 [cited 2022 Aug 3]. Available from: <https://www.ons.gov.uk/peoplepopulationandcommunity/birthsdeathsandmarriages/deaths/bulletins/deathsregistrationsummarytables/2020>

3. Owen-Williams R. Leading causes of death, UK - Office for National Statistics [Internet]. www.ons.gov.uk. 2020 [cited 2022 Aug 3]. Available from: <https://www.ons.gov.uk/peoplepopulationandcommunity/healthandsocialcare/causesofdeath/articles/leadingcausesofdeathuk/2001to2018>

4. Kauvar DS, Sarfati MR, Kraiss LW. National trauma databank analysis of mortality and limb loss in isolated lower extremity vascular trauma. Journal of vascular surgery. 2011 Jun 1;53(6):1598-603.

5. Perkins ZB, De'Ath HD, Aylwin C, Brohi K, Walsh M, Tai NR. Epidemiology and outcome of vascular trauma at a British Major Trauma Centre. European journal of vascular and endovascular surgery. 2012 Aug 1;44(2):203-9.

6. Kobayashi L, Coimbra R, Goes Jr AM, Reva V, Santorelli J, Moore EE, Galante J, Abu-Zidan F, Peitzman AB, Ordonez C, Maier RV. American Association for the Surgery of Trauma–World Society of Emergency Surgery guidelines on diagnosis and management of peripheral vascular injuries. Journal of Trauma and Acute Care Surgery. 2020 Dec 1;89(6):1183-96.

7. Demetriades D, Velmahos GC, Scalea TM. Blunt Traumatic Thoracic Aortic Injuries: Early or Delayed Repair—Results of an American Association for the Surgery of Trauma Prospective Study. Journal of Vascular Surgery. 2010 Oct 1;52(4):1111-2.

8. Fox N, Schwartz D, Salazar JH, Haut ER, Dahm P, Black JH, Brakenridge SC, Como JJ, Hendershot K, King DR, Maung AA. Evaluation and management of blunt traumatic aortic injury. Journal of Trauma Nursing. 2015 Mar 1;22(2):99-110.

9. Fox N, Rajani RR, Bokhari F, Chiu WC, Kerwin A, Seamon MJ, Skarupa D, Frykberg E. Evaluation and management of penetrating lower extremity arterial trauma: an Eastern Association for the Surgery of Trauma practice management guideline. Journal of Trauma and Acute Care Surgery. 2012 Nov 1;73(5):S315-20.

10. Tisherman SA, Bokhari F, Collier B, Ebert J, Holevar M, Cumming J, Kurek S, Leon S, Rhee P. Clinical practice guidelines: penetrating neck trauma. Chicago (IL): Eastern Association for the Surgery of Trauma (EAST). 2008.

11. Arrillaga A. Practice management guidelines for penetrating trauma to the lower extremity. EAST practice management work group. Eastern Association for the Surgery of Trauma. 2000.

12. TARN (2021b). TARN Analytics User Guide. [online] Available at:

https://www.tarn.ac.uk/content/downloads/53/TARN%20Analytics%20Guidance%20June%2

021.pdf [Accessed 24 Oct. 2021].

13. Benson RA, Nandhra S. Outcomes of vascular and endovascular interventions performed during the coronavirus disease 2019 (COVID-19) pandemic: the vascular and endovascular research network (VERN) Covid-19 vascular service (COVER) tier 2 study. Annals of surgery. 2021 Apr;273(4):630.

14. Groin wound Infection after Vascular Exposure (GIVE) Study Group, Gwilym BL, Saratzis A, Benson RA, Forsythe R, Dovell G, Dattani N, Lane T, Shalhoub J, Bosanquet D, Gwilym BL. Groin wound infection after vascular exposure (GIVE) multicentre cohort study. International Wound Journal. 2021 Apr;18(2):164-75.

15. Marsden ME, Vulliamy PE, Carden R, Naumann DN, Davenport RA, Nnajiuba HO, Perkins Z, Pearce P, Yalamanchili S, Malik NS, Tai N. Trauma laparotomy in the UK: A prospective national service evaluation. Journal of the American College of Surgeons. 2021 Sep 1;233(3):383-94.

16. EuroSurg Collaborative, Sgrò A, Blanco‐Colino R, Ahmed WU, Brindl N, Gujjuri RR, Lapolla P, Mills EC, Pérez‐Ajates S, Soares AS, Van Straten S. Management of COMPlicAted intra‐abdominal collectionS after colorectal Surgery (COMPASS): protocol for a multicentre, observational, prospective international study of drain placement practices in colorectal surgery. Colorectal Disease. 2020 Dec;22(12):2315-21.

17. Kouli O, Chaudhry D, Shafi SQ, Riad AM, Bhangu A, Biccard B, Blanco-Colino R, Docherty AB, El-Boghdadly K, Glasbey JC, Harrison EM. CArdiovaSCulAr outcomes after major abDominal surgEry: study protocol for a multicentre, observational, prospective, international audit of postoperative cardiac complications after major abdominal surgery. British Journal of Anaesthesia. 2022 May 1;128(5):e324-7.

18. Medical Research Council. Is my study research? [Internet]. Hra-decisiontools.org.uk. 2019 [cited 2022 Aug 13]. Available from: <http://www.hra-decisiontools.org.uk/research/>

19. Medical Research Council. Do I need NHS Ethics approval? [Internet]. www.hra-decisiontools.org.uk. 2020 [cited 2022 Aug 13]. Available from: <http://www.hra-decisiontools.org.uk/ethics/>

**Supplementary Figures and Tables**

**Table S1 – Complete list of datapoints to be collected (page 3)**

| **Data Field** | **Data Specifics** |
| --- | --- |
| Patient Demographics | Sex  Age  Ethnicity  Comorbidities – *smoking status, diabetes, hypertension, ischaemic heart disease, chronic kidney disease, peripheral vascular disease* |
| Injury Data | Type of injury – blunt vs penetrating  Mechanism of injury  Injury sustained  Any associated injuries?  Abbreviated injury scale  Injury severity score  Observations and blood gas on arrival in ED  Prehospital + ED intervention – *tourniquet, blood products, TXA, thoracostomy/ thoracotomy*  Grade of shock on arrival in ED |
| Site Data | Location of presenting hospital – rural or urban?  Transferred out?  Transfer destination (vascular centre, MTC)  Time from presentation to transfer  Reason for transfer + any delay |
| Management Data | Method of diagnosis – imaging modality vs surgery  Time from presentation to intervention  Intervention performed  Most senior grade of surgeon  Primary operator speciality |
| Operative Findings | Record anatomical level of injury  Confirm injury sustained  Materials used in repair  Primary amputation? |
| Outcome Data | Total length of stay  Length of critical care stay  Death at 30 days  Amputation rate at 30 days  Complications  Return to theatre  Discharge destination |
